# Behind the Lens: A Cross-Sectional Study of Convergence Insufficiency in Photographers

**DOI:** 10.7759/cureus.85012

**Published:** 2025-05-29

**Authors:** Manda Nirmala Jyothi, Gurunadh S Velamakanni, Kandula Satish, Manne Sri Hari Babu, B V Satyanarayana

**Affiliations:** 1 Ophthalmology, GSL Medical College, Rajahmundry, IND; 2 General Medicine, GSL Medical College, Rajahmundry, IND

**Keywords:** age, ci-convergence insufficiency, ciss-convergence insufficiency symptom survey, photographers, working hours

## Abstract

Introduction: Convergence insufficiency (CI) is a binocular vision disorder affecting near focus, often impacting professionals with high visual demands, such as photographers. Symptoms include eye strain, fatigue, blurred vision, headaches, and difficulty concentrating. This study investigates CI prevalence among photographers using the Convergence Insufficiency Symptom Survey (CISS) questionnaire, aiming to facilitate early detection, clinical evaluation, and intervention strategies to optimize visual performance and well-being.

Materials and methods: This cross-sectional, descriptive study was conducted at the Department of Ophthalmology, GSL Medical College, Rajahmundry, using a survey-based approach with convenience sampling. Participants (aged 18-43) had ≥1 year of photography experience and ≥4 hours of daily work. Exclusions included uncorrected refractive errors, ocular deviations, and neuromuscular disorders. Data analysis involved descriptive statistics, regression, and Pearson correlation. Ethical approval was obtained.

Results: A total of 52 male participants (mean age ± SD: 25.9 ± 6.2 years; range: 18-43 years) were included in the study; no female participants were enrolled. The mean CISS score was 21.6 ± 5.1 (range: 12-33). None of the participants scored within the 0-10 range. Moderate symptoms (scores: 21-30) were reported by 25 participants (48%), mild symptoms (scores: 11-20) by 23 participants (44%), and severe symptoms (scores: >31) by four participants (7.7%). A moderate positive correlation was observed between age and CISS scores (r = 0.51, R² = 0.26, p < 0.001). In contrast, work hours (WH) showed a weak negative correlation (r = -0.26, R² = 0.07, p = 0.05). Analysis of variance (ANOVA) (F = 3.87) suggested a weak overall effect. Severe symptoms were predominantly found in participants older than 36 years, despite them reporting shorter durations of near work.

Conclusion: This study demonstrated a notable association between age, working hours, and the severity of CI symptoms among professional photographers. Younger participants exhibited mild to moderate symptoms despite long WH, whereas older individuals reported more severe symptoms even with less WH. These findings emphasize the importance of early visual assessments and targeted interventions for professional photographers to improve visual health and performance.

## Introduction

Convergence insufficiency (CI) is a common binocular vision disorder in which the eyes struggle to work together effectively when focusing on near objects. This condition, often associated with visual tasks that require sustained near vision, such as reading or using digital devices, can have profound implications for individuals whose work demands precise visual coordination [1]. Photographers, for instance, rely heavily on their visual accuracy when setting up their equipment, reviewing images, and adjusting camera settings, tasks that frequently require a sharp focus on near objects for longer durations.

However, individuals with CI may experience significant challenges in performing these tasks, leading to common symptoms such as eye strain, fatigue, blurred vision for near, headache, diplopia for near, sensation of print moving while reading, difficulty with reading comprehension, with symptom that present after short period of reading or prolonged near work being sleepiness and inability to concentrate [2]. As a profession that demands sustained, precise visual focus, the impact of CI on the work performance of photographers and overall well-being is a critical yet underexplored area. The prevalence of CI in photographers, its potential effects on their visual performance, and the strategies for addressing the condition are key topics that require further investigation. Although CI has been extensively studied in other fields, its impact on photographers remains largely unexplored. This study aims to address this gap by investigating the prevalence of CI symptoms among photographers by using the Convergence Insufficiency Symptom Survey (CISS) [3] questionnaire. The study would also help in advising the participants on further clinical investigations and potential therapies or treatment options to overcome the symptoms.

## Materials and methods

It was a cross-sectional, descriptive research design, conducted at the Department of Ophthalmology, GSL Medical College, Rajahmundry. A survey-based approach was used to gather data, participants were selected through a convenience sampling method from January 2, 2025, to April 3, 2025. Minimum one year of professional photography experience, at least four hours work per day, age between 18 and 43 years, and regular performance of near-vision tasks, such as editing photos on digital screens and using digital single-lens reflex (DSLR) camera displays at close range distance, were included in this study.

Individuals with uncorrected refractive errors, anisometropia, ocular deviations, diplopia, and nystagmus were excluded from the study. Additionally, subjects with systemic diseases affecting neuromuscular function and vision, such as Parkinson's disease, multiple sclerosis, myasthenia gravis, and thyroid eye disease, were excluded. Those using corrective interventions for CI, like plus-add lenses and base-in prisms, were also excluded.

Ocular and general examinations were performed. After obtaining informed consent, the CISS was administered to assess the presence and severity of CI symptoms. The questionnaire was given and explained by a single person in two languages: English and the participant’s mother tongue, Telugu. The questionnaire was administered by a single examiner to all the study participants. According to Rouse et al. [3] and Nunes et al. [4], the CISS is a standard tool for screening CI symptoms in adults with normal binocular vision (NBV). It is also a standard tool to assess CI symptoms among presbyopic adults [5]. The CISS comprises 15 questions, each with five response options: never (0), infrequently/not very often (1), sometimes (2), fairly often (3), and always (4). The total CISS score for each participant was calculated by summing the scores of all 15 items. A score of 21 or greater on the CISS was considered indicative of symptoms [3]. Specifically designed to evaluate symptoms commonly associated with CI, such as eye strain, headache, blurred vision, double vision, and difficulty focusing during sustained near tasks, participants rated the frequency and intensity of these symptoms on a scale ranging from 0 (never) to 4 (always). Participants were asked to answer the survey questions based on their experiences during extended near-vision activities that are typical in professional photography. The severity of CI symptoms was classified based on the total CISS score: 0-10 indicating no symptoms, 11-20 mild symptoms, 21-30 moderate symptoms, and 31-60 severe symptoms. 

Data collected from the CISS questionnaire were analyzed using MS Excel (Microsoft Corporation, Redmond, Washington, United States). The frequency of reported symptoms was calculated, and the total CISS scores for each participant were used to categorize the severity of CI. Microsoft Office Excel and IBM SPSS Statistics for Windows, Version 20 (Released 2011; IBM Corp., Armonk, New York, United States) were used to compute descriptive statistics, including the mean, standard deviation, and frequency distribution. Regression analysis and the Pearson correlation coefficient were used to calculate the statistical significance. A p-value of <0.05 is considered statistically significant. 

Ethical approval for the study was obtained from the Institutional Ethical Board (IEB). All participants were provided with detailed information about the purpose of the study. The confidentiality of the participants was maintained throughout the study.

## Results

A total of 52 participants were included in this study, and all the participants were male, with ages ranging from 18 to 43 years (mean ± SD: 25.9 ± 6.2). The CISS scores ranged from 12 to 33, with a mean score of 21.6 ± 5.1. The participants' work hours (WH) varied between four and 15 hours, with a mean of 8.6 ± 3.5 hours (Table 1).

**Table 1:** Descriptive statistics CISS: Convergence Insufficiency Symptom Survey

Parameter	Minimum	Maximum	Mean ± SD
Age (years)	18	43	25.9 ± 6.2
CISS score	12	33	21.67 ± 5.1
Work hours per day	4	15	8.6 ± 3.5

The CISS score of the participants was analyzed (Table 2). Notably, no participants scored within the 0-10 CISS score. Majority of the participants (25/52, 48.10%) had a CISS score between 21 and 30. Of the remaining, 23/52 (44.20%) had a score between 11 and 20, and only 4/52 (7.70%) had a score of >31.

**Table 2:** Age and CISS score comparison among the study members; n (%) CISS: Convergence Insufficiency Symptom Survey

Age (years)	CISS score			Total	Statistical analysis
	11-20	21-30	>31		
<20	5 (31.3)	11 (68.8)	0	16 (100)	Chi-square value = 44.9; p-value = 0.001
21-25	10 (76.9)	2 (15.4)	1 (7.7)	13 (100)	
26-30	8 (61.5)	5 (38.5)	0	13 (100)	
31-35	0	6 ((100)	0	6 (100)	
36-40	0	1 (33)	2 (66.7)	3 (100)	
>41	0	0	1 (100)	1 (100)	
Total	23 (44.2)	25 (48.1)	4 (7.7)	52 (100	

**Figure 1:**
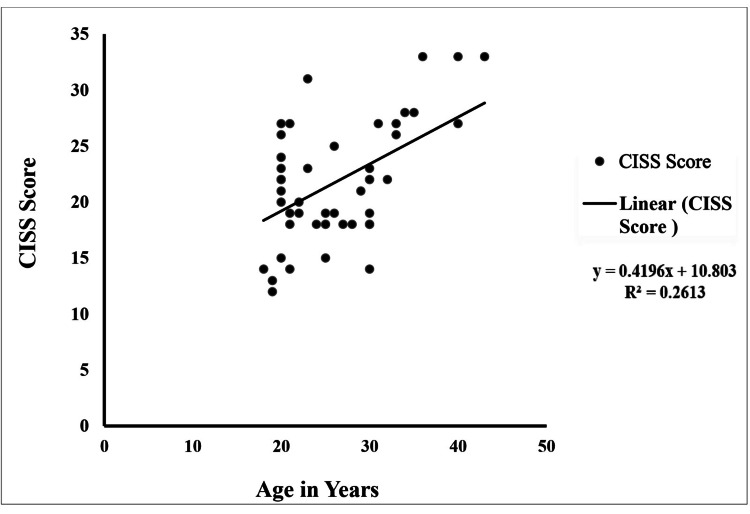
Correlation between the age and the CISS score CISS: Convergence Insufficiency Symptom Survey

A moderate positive correlation (r = 0.51) was observed between age and CISS score, indicating that as age increases, the CISS score tends to increase. The analysis explained 26% of the variance in the CISS score (R² = 0.26) and was statistically significant (p < 0.001). The F-value of 17.9, obtained from ANOVA (Table 2), further supports the significance of the relationship (Figure 1).

The majority of participants (23/52) working 6-10 hours per day included 13 (56.00%) with moderate CISS scores (21-30) and 10 (43.50%) with mild scores (11-20) (Table 3). Among the 13 participants working 1-5 hours, six (46.20%) had moderate symptoms, four (30.80%) had mild symptoms, and three (23.10%) had severe symptoms. The Chi-square value for WH and CISS score was 7.59, with a p-value of 0.18, indicating no significant association.

**Table 3:** Work hours (WH) and CISS score comparison among the study members; n (%) CISS: Convergence Insufficiency Symptom Survey

WH	CISS score			Total	Statistical analysis
	11-20	21-30	>31		
1-5	4 (30.8)	6 (46.2)	3 (23.1)	13 (100)	Chi-square value = 7.59; p-value = 0.18
6-10	10 (43.5)	13 (56.5)	0	23 (100)	
11-15	9 (56.3)	6 (37.5)	1(6.3)	16 (100)	
Total	23 (44.2)	25 (48.1)	4 (7.7)	52 (100)	

**Figure 2:**
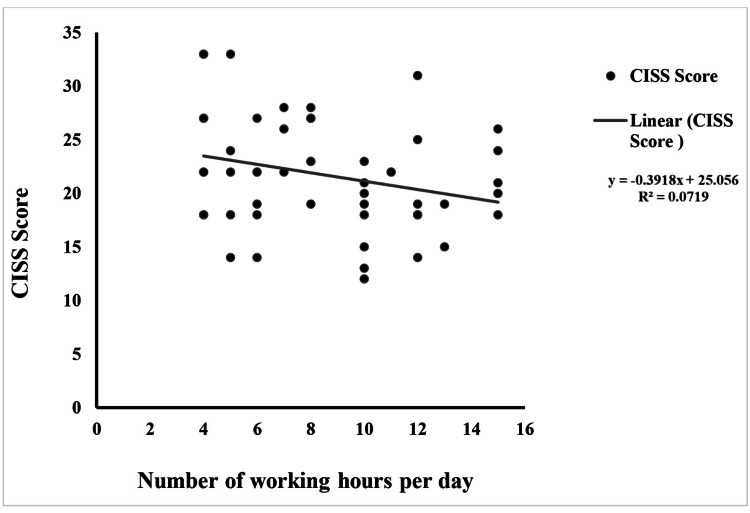
Correlation between work hours and CISS score CISS: Convergence Insufficiency Symptom Survey

The correlation between WH and CISS score was weakly negative (r = -0.26) with a p-value of 0.05, indicating a borderline significant relationship (Figure 2). The R-squared value was 0.07, suggesting that WH explained only 7% of the variance in CISS scores. The ANOVA test showed an F-value of 3.87, indicating a weak effect of WH on the CISS scores.

The distribution of CISS scores among photographers across different age and working hours (WH) per day was presented in Table 3. Most participants aged <30 reported mild (11-20) to moderate (21-30) symptoms. Severe symptoms (>31) were found in only four: one aged 21-25 (WH 11-15), two aged 36-40 (WH 1-5), and one aged >41 (WH 1-5). The 21-25 and 26-30 age groups showed the highest frequency of mild to moderate CI symptoms, those working longer hours (11-15/day) (Table 4). 

**Table 4:** Age and WH comparison with CISS score; n (%) WH: work hours per day; CISS: Convergence Insufficiency Symptom Survey

Age (years)	Work hours per day	CISS score			Total
		11-20	21-30	>31	(52): n (%)
	1-5	0	3 (100)	0	3 (100)
<20	6-10	3 (50)	3 (50)	0	6 (100)
	11-15	2 (28.6)	5 (71.4)	0	7 (100)
	1-5	1 (50)	1 (50)	0	2 (100)
21-25	6-10	4 (80)	1 (20)	0	5 (100)
	11-15	5 (83.3)	0	1 (16.7)	6 (100)
	1-5	3 (100)	0	0	3 (100)
26-30	6-10	3 (42.9)	4 (57.1)	0	7 (100)
	11-15	2 (66.7)	1 (33.3)	0	3 (100)
	1-5	0	2 (100)	0	2 (100)
31-35	6-10	0	4 (100)	0	4 (100)
	11-15	0	0	0	0
	1-5	0	0	2 (100)	2 (100)
36-40	6-10	0	1 (100)	0	1 (100)
	11-15	0	0	0	0
>41	1-5	0	0	1 (100)	1 (100)
	6-10	0	0	0	0
	11-15	0	0	0	0

## Discussion

The main objective of this study was to screen for CI symptoms among professional photographers using the CISS questionnaire. The study analyzed a total of 52 participants, and all the participants were male. In the present study, 29 participants (55.76%) scored >21, thereby indicating that most of the participants exhibit symptomatic manifestations. The mean CISS score was 21.6 + 5.1. The range of scores is presented in Table 2. According to Darko Takyi et al., the CISS score is associated with both the severity and the frequency of signs of CI in young adults [6].

Ghadban et al. [7] and Pickwell [8] observed a notable increase in the incidence of CI with advancing age. Consistent with these findings, the current study found a positive correlation (r = 0.51) between age and the CISS score, with a statistical significance (p = 0.0001).

When the CISS scores were analyzed with age and WH, a mild score (14) was observed with 12 hours of work for an 18-year-old individual, whereas it was a severe score (33) for a 43-year-old participant with four WH. Statistically, there was a negative correlation (r = -0.26) between work duration and CISS score. This suggests that as age increases, the mean CISS score also increases. As age increases, a decrease in the number of working hours is observed, accompanied by an increase in the CISS score. Duration of working hours is not directly related to the CISS score. A weak negative correlation (r = -0.26, p = 0.05) between CISS score and working hours suggests other factors may be influencing CISS scores more strongly. Working atmosphere, stress in the work environment, and so on may be responsible, but these were not considered in this study.

CISS scores were analyzed across age and WH. Notably, none of the participants scored below 10, indicating that all exhibited at least mild symptoms. The most prevalent symptom range fell between scores of 11 and 30, representing mild to moderate CI symptoms. In the <20 years group, all reported moderate symptoms (21-30), especially those with 1-5 WH per day. As working hours increased, a mix of mild and moderate symptoms was seen, 50% (3) of the participants with 6-10 WH reported mild symptoms, and the remaining 50% (3) reported moderate symptoms. In those working 11-15 hours, moderate symptoms dominated (71.4%; 5). This trend is consistent with the findings of Rouse et al., who reported increased CI symptoms with prolonged near work, especially among younger individuals such as school-aged children and students involved in high levels of visual demand [9].

The 21-25 and 26-30 groups demonstrated a high proportion of mild and moderate symptoms as well. Among participants aged 21-25, a high percentage (80-83.3%) working 6-15 hours had mild symptoms, while those working fewer hours had equal distribution between mild and moderate symptoms. One participant in the 11-15 hour group in this age bracket exhibited severe symptoms (>31), aligning with the notion that increased duration of near-vision tasks may exacerbate CI symptoms. These findings are supported by the work of Scheiman et al., who emphasized the impact of continuous near work in digital environments on symptom severity in adults [10].

In the 26-30 age group, individuals working fewer hours (1-5) showed exclusively mild symptoms, whereas those working 6-15 hours showed a shift toward moderate symptoms. Interestingly, no severe symptoms were observed in this age category. This pattern aligns with the study by Yaramothu et al., which found that moderate symptoms were more common among young adults engaging in intermediate durations of near tasks, with severity increasing only after a certain threshold of work duration [11].

For participants aged 31-35, only moderate symptoms were observed, and only among those working 1-10 hours. Those working 11-15 hours did not report any symptoms, which may suggest either a small sample size or adaptation mechanisms at play. Conversely, in the 36-40 and >41 age groups, fewer participants were involved, but notable cases of severe symptoms were recorded, especially among those working fewer hours (1-5). This is a unique finding and contrasts with the general pattern of higher symptom scores correlating with longer WH. This deviation might be better understood in the literature. It was reported that age-related changes in fusional reserves and accommodative flexibility can lead to increased symptom severity even with shorter near work durations in older adults [12].

Overall, this data highlights a strong relationship between working hours, age, and CI symptom severity among photographers. Young adults and middle-aged individuals showed a concentration of mild-to-moderate symptoms, particularly with longer near-vision tasks. However, severe symptoms in older age groups with shorter working durations suggest a complex interplay of age-related ocular physiology and visual demands. Further investigation with larger and more diverse samples could offer greater insight into the occupational risks of CI in visual-intensive professions.

This study aims to help photographers enhance both the quality of their work and their visual health, enabling them to perform at their best. By raising awareness of CI and providing strategies for managing the condition, it seeks to improve their overall well-being and work efficiency.

Limitations 

This study has a few limitations. The sample size was small and included only male participants, which may limit the generalizability of the findings to the wider population. Although the CISS is a validated symptom-based tool, the absence of objective clinical measures such as near point of convergence and fusional vergence may limit diagnostic accuracy. Additionally, factors like working posture and occupational stress, work ergonomics, screen time variability, and psychosocial stress were not evaluated, though they may influence the severity of symptoms. Future studies should include objective testing and a more diverse sample.

## Conclusions

This study highlights that 55.8% (29/52) of professional photographers exhibited moderate to severe CI symptoms, with a mean CISS score of 21.6 ± 5.1 (range: 12-33). Age showed a moderate positive correlation with symptoms (r = 0.51, p < 0.001), while WH had a weak negative correlation (r = -0.26, p = 0.05) with no significant association (Chi-square = 7.59, p = 0.18). Younger participants mostly experienced mild to moderate symptoms, whereas severe symptoms were more common among older individuals with fewer WH. These findings emphasize the impact of age and WH on CI symptoms in photographers. Early screening and clinical evaluation are essential for effective management.

## Figures and Tables

**Figure 3:**
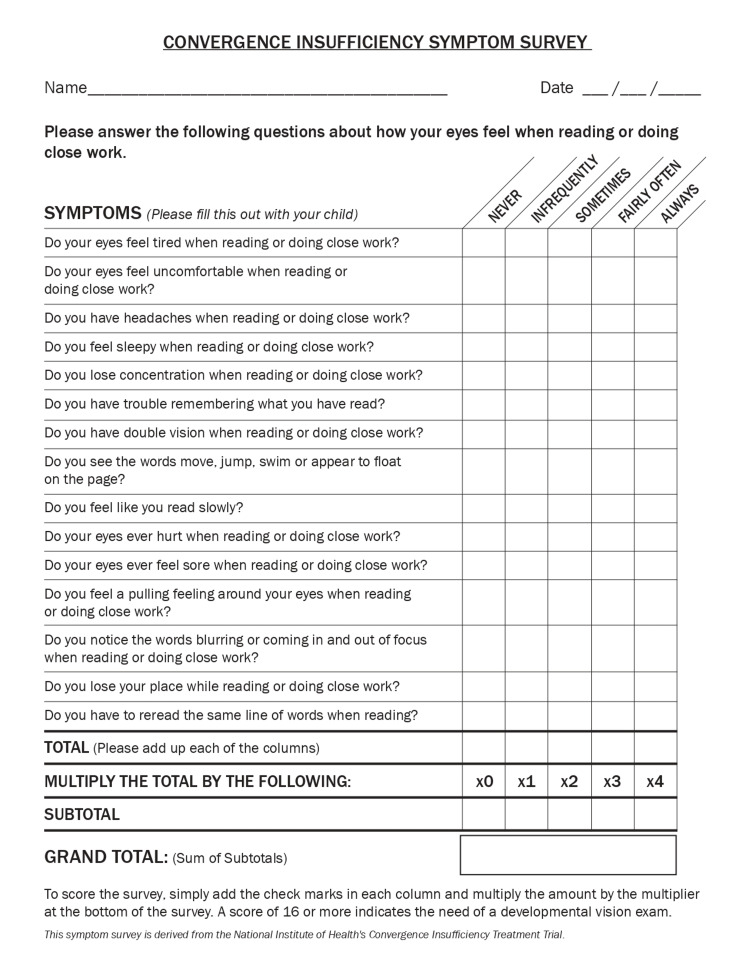
Convergence Insufficiency Symptom Survey Questionnaire
